# Preliminary Study of Resistance Mechanism of *Botrytis cinerea* to SYAUP-CN-26

**DOI:** 10.3390/molecules27030936

**Published:** 2022-01-29

**Authors:** Kai Wang, Huazhong Zhang, Wanying Zhu, Jingnan Peng, Xinghai Li, Yingzi Wang, Zhiqiu Qi

**Affiliations:** Department of Pesticide Science, Plant Protection College, Shenyang Agricultural University, Shenyang 110866, China; wangkai12@syau.edu.cn (K.W.); zhz961116@163.com (H.Z.); wanyingzhu1998@outlook.com (W.Z.); pjn941106@163.com (J.P.); xinghai30@163.com (X.L.); wangyz3333@163.com (Y.W.)

**Keywords:** SYAUP-CN-26, *Botrytis cinerea*, activity, cell membrane

## Abstract

SYAUP-CN-26 (1*S*, 2*R*-((3-bromophenethyl)amino)-*N*-(4-chloro-2-trifluoromethylphenyl) cyclohexane-1-sulfonamide) is a novel sulfonamide compound with excellent activity against *Botrytis cinerea*. The present study sought to explore the mutant of *B.* *cinerea* resistant to SYAUP-CN-26 using SYAUP-CN-26 plates. Moreover, the cell membrane functions of *B.* *cinerea*, histidine kinase activity, relative conductivity, triglyceride, and cell membrane structure were determined, and the target gene histidine kinase Bos1 (AF396827.2) of procymidone was amplified and sequenced. The results showed that compared to the sensitive strain, the cell membrane permeability, triglyceride, and histidine kinase activity of the resistant strain showed significant changes. The relative conductivity of the sensitive strain increased by 6.95% and 9.61%, while the relative conductivity of the resistant strain increased by 0.23% and 1.76% with 26.785 µg/mL (EC_95_) and 79.754 µg/mL (MIC) of SYAUP-CN-26 treatment. The triglyceride inhibition rate of the resistant strain was 23.49% and 37.80%, which was 0.23% and 1.76% higher than the sensitive strain. Compared to the sensitive strain, the histidine kinase activity of the resistant strain was increased by 23.07% and 35.61%, respectively. SYAUP-CN-26 significantly damaged the cell membrane structure of the sensitive strain. The sequencing of the Bos1 gene of the sensitive and resistant strains indicated that SYAUP-CN-26 resistance was associated with a single point mutation (P348L) in the Bos1 gene. Therefore, it was inferred that the mutant of *B.* *cinerea* resistant to SYAUP-CN-26 might be regulated by the Bos1 gene. This study will provide a theoretical basis for further research and development of sulfonamide compounds for *B. cinerea* and new agents for the prevention and control of resistant *B. cinerea*.

## 1. Introduction

*Botrytis cinerea*, an airborne, necrotrophic plant pathogen infecting over 200 plant species, including essential oil, fiber, and horticultural crops, is the causative agent of gray mold [1]. Gray mold is characterized by the rot of flowers, leaves, and fruits of tomatoes and other vegetables, resulting in significant yield losses [2,3,4,5]. Until now, multiple applications of fungicides, such as benzimidazoles, dicarboximides, succinate dehydrogenase inhibitors, anilinopyrimidine, and quinone outside inhibitors, have remained the only effective approaches to control this disease due to the absence of resistant varieties to this pathogen. However, the persistent use of these fungicides has developed and increased multiple resistances in *B. cinerea*, leading to the failure to control gray mold [6,7,8,9,10,11]. Therefore, developing novel fungicides with unique structures and mechanisms for controlling resistant strains has become highly necessary. 

Pesticides designed with sulfonamide derivatives have excellent antifungal activity [12,13], such as flusulfamide. Flusulfamide is the first sulfonamide fungicide developed and marketed by the Japan Mitsui Toatsu Co., Ltd. in 1986 as a soil fungicide for the prevention of *Plasmodiophora brassicae* Woronin [14]. Similarly, tolnifanide was developed by the Sumitomo Corporation of Japan for preventing and treating *Cladosporium cucumerinum*, while amisulbrom was developed by Japan Nissan Chemical Co. Ltd. (Tokyo, Japan) for preventing and treating vegetable downy mildew [15]. Cyazofamid, developed by Ishihara Sangyo Kaisha Ltd., is a protective fungicide for the prevention and control of oomycetes [16]. Recently, researchers have synthesized dozens of sulfonamide-based active compounds [17,18,19] containing polycyclic alkyl groups but with different antifungal activities from cyazofamid, amisulbrom, and tolnifanide. Ezabadi et al. synthesized Sulfonamide-1 and 2, 4-triazole derivatives with outstanding activity against *B. cinerea*, *Aspergillus flavus*, and *Trichoderma viride* [17]. Wang et al. developed a sulfonamide associated with the cycloalkyl groups mostly active against *Gibberella zeae* and *Venturia nashicola* [18]. Yang et al. found a cycloalkyl suflonamide compound, showing excellent antifungal activity against *Rhizoctonia solani*, *B. cinerea,* and *Sclerotinia sclerotiorum* [19]. Accumulating studies have reported that the mechanism of action of cycloalkyl suflonamide compounds might be different from commercially available fungicides [20,21]. 

**Figure 1:**
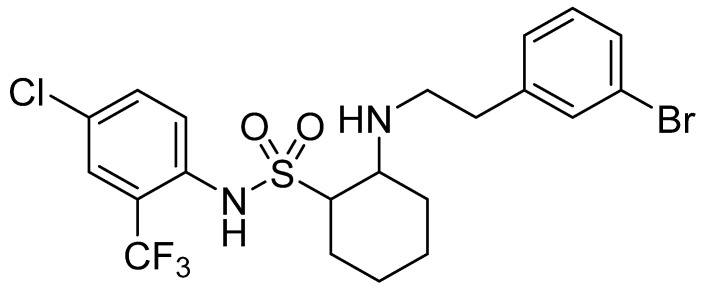
Structure of SYAUP-CN-26 (1*S*, 2*R*-((3-bromophenethyl) amino)-*N*-(4-chloro-2-(trifluoromethyl) phenyl) cyclohexane-1-sulfonamide).

SYAUP-CN-26 (used in this study), chemically named (1*S*, 2*R*-((3-bromophenethyl)amino)-*N*-(4-chloro-2-(trifluoromethyl)phenyl)cyclohexane-1-sulfonamide), is a cycloalkyl compound containing one sulfonyl group with significant activity against *B. cinerea* [22,23,24,25] (Figure 1). A previous study reported that SYAUP-CN-26 could affect the mitochondrial and cell membrane’s structure and function of *B. cinerea* [25,26]. Meanwhile, cross-resistance between SYAUP-CN-26 and procymidone was observed against the sensitivity of *B. cinerea* strains (Table S1, Table S2 and Figure S1 in Supplementary Material), indicating that SYAUP-CN-26 might possess a similar action mechanism as procymidone against *B. cinerea*, and procymidone would lead to lipid peroxidation, inhibit triglyceride synthesis and mycelial growth, and damage the cell membrane. Researchers have found that the mutation of Bos1 would lead to the resistance of *B. cinerea* to procymidone [27,28,29]. Therefore, this study assessed the electrical conductivity, cell membrane permeability, triglyceride content, and Bos1 sequence of the resistant and sensitive strains of *B. cinerea* to SYAUP-CN-26 to understand the mechanism of action of SYAUP-CN-26 and infer the action sites of SYAUP-CN-26.

## 2. Results

### 2.1. Generation of B. cinerea Mutant Resistance to SYAUP-CN-26

One SYAUP-CN-26-resistant mutant was obtained after repeated exposure to SYAUP-CN-26. This strain grew well on PDA plates amended with 200 µg/mL of SYAUP-CN-26, while its parent could not grow. The resistance level RF values were 69.2230 and 69.9010 with 1st and 10th generations of the *B. cinerea* mutant. The mutant showed high resistance to SYAUP-CN-26. After 10 generations of transfer, the sensitivity change factor FSC value of the strain was 1.0098. FSC ≈ 1 was considered as the threshold when resistance of the mutant could be inherited stably (Table 1).

### 2.2. Effect of SYAUP-CN-26 on Cell Membrane

**Figure 2:**
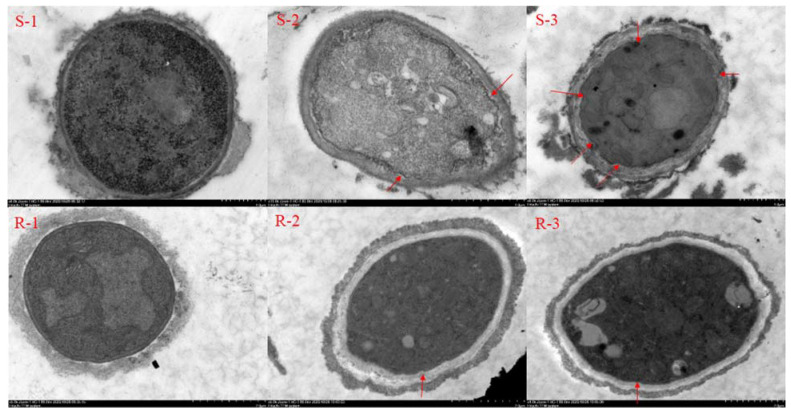
Effect of SYAUP-CN-26 on *B. cinerea* cell membrane. S—sensitive strain; R—resistant strain; R-1: resistant control; S-1: sensitive control; 2—26.758 µg/mL; 3—79.754 µg/mL, red arrow: cell membrane fold.

**Figure 3:**
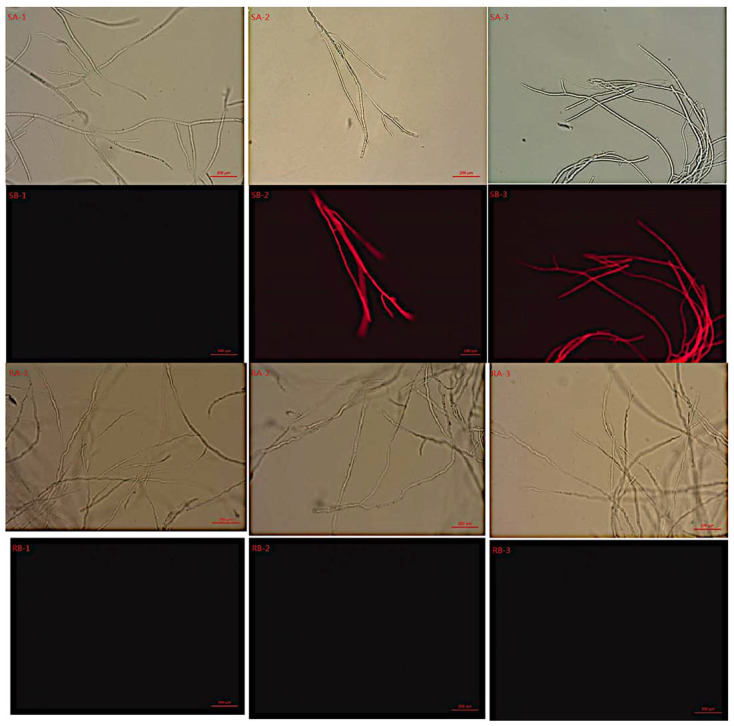
Effects of SYAUP-CN-26 on *B. cinerea* cell membrane with fluorescence microscope. S—sensitive strain; R—resistant strain; 1—control; 2—26.758 µg/mL; 3—79.754 µg/mL; A—optical electron microscope; B—fluorescence microscope.

The cell membrane and transmission electron microscope (Figure 2) results showed that when the sensitive strain was treated without SYAU-CN-26, the cell membrane of mycelium was complete and showed a clear structure (Figure 2 S-1). The mycelial membrane structure was gradually destroyed with the increase of SYAUP-CN-26 concentration, and more folds appeared. (Figure 2 S-2, S-3). When the concentration reached 79.754 µg/mL (MIC), the cell membrane structure was severely damaged (Figure 2 S-3). When the resistant strain was not treated with SYAU-CN-26, the mycelial cell membrane was concave inward (Figure 2 R-1). With the increase of SYAUP-CN-26 concentration, the degree of inward depression of the mycelium of the drug-resistant strain changed a little compared to the blank control (Figure 2 R-2). When the concentration reached 79.754 µg/mL, the cell membrane of the resistant strain was also folded (Figure 2 R-3).

The sensitive and resistant strains without SYAUP-CN-26 treatment did not show fluorescence under the fluorescence microscope (Figure 3 SB-1, RB-1). However, the sensitive mycelium treated with 26.758 and 79.754 µg/mL SYAUP-CN-26 showed fluorescence (Figure 3 SB-2, SB-3), while the resistant mycelium did not show fluorescence (Figure 3 RB-2, RB-3). 

### 2.3. Measurement of Relative Electric Conductivity

**Figure 4:**
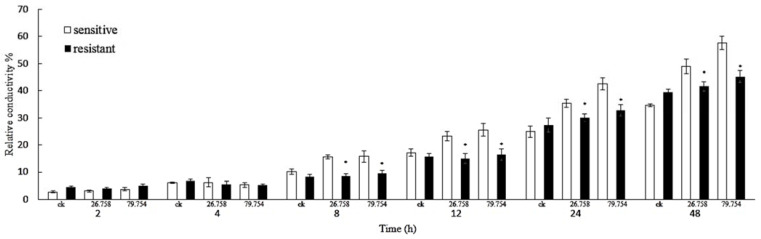
Relative electric conductivity of *B. cinerea* with SYAUP-CN-26. Values are presented as mean ± S.E. (n = 3) and are significant for * *p* < 0.05.

SYAUP-CN-26 could change the cell membrane permeability of the sensitive strain but had little effect on the cell membrane permeability of the resistant strain (Figure 4). After 12 h of treatment, the relative conductivity of the sensitive and resistant strains showed a significant difference (*p* < 0.05) of 22.10% and 17.49% with 26.785 µg/mL SYAUP-CN-26 and 22.10% and 17.49% with 79.754 µg/mL, respectively. After 48 h, the average relative conductivity of the sensitive strain increased by 6.95% and 9.61%, respectively, and the resistance strain increased by 0.23% and 1.76% with 26.785 µg/mL and 79.754 µg/mL SYAUP-CN-26, respectively, compared with the control. 

### 2.4. Effect of SYAUP-CN-26 on the Triglyceride Synthesis (TG) of B. cinerea

**Figure 5:**
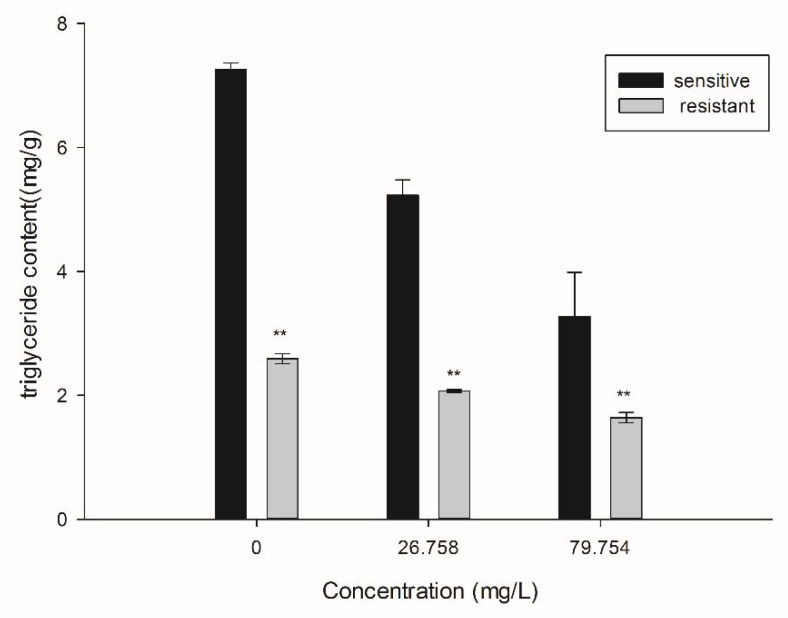
Effect of SYAUP-CN-26 on the triglyceride synthesis (TG) of *B. cinerea.* Values are presented as mean ± S.E. (n = 3) and are significant for ** *p* < 0.01.

SYAUP-CN-26 could effectively inhibit triglyceride synthesis in the mycelium of *B. cinerea*, and the inhibition rate of triglyceride synthesis of the sensitive strain was higher than that of the resistant strain (Figure 5). The inhibition rates of triglyceride content of the sensitive and resistant strains increased from 27.93% and 20.26% at 26.758 µg/mL SYAUP-CN-26 to 55% and 36.68% at 79.754 µg/mL, respectively.

### 2.5. Detection of Histidine Kinase (HK) Activity

**Figure 6:**
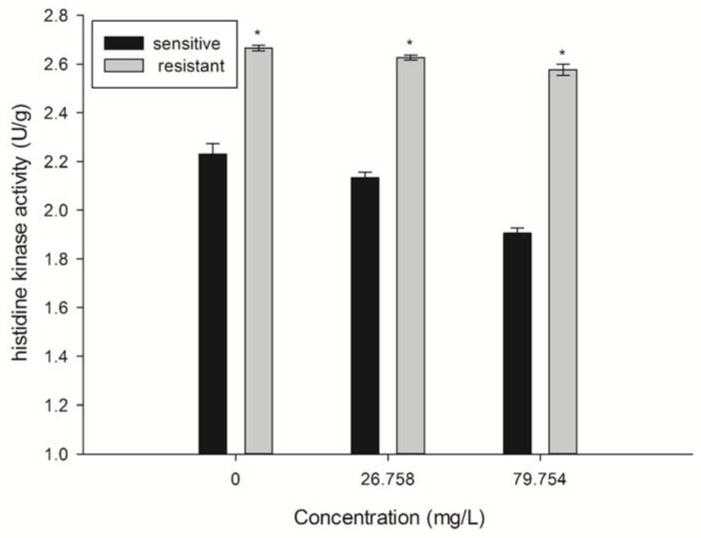
The histidine kinase (HK) activity of *B. cinerea* with SYAUP-CN-26. Values are presented as mean ± S.E. (n = 3) and are significant for * *p* < 0.05.

There were significant differences in the histidine kinase activity of the sensitive and resistant strains (*p* < 0.05) (Figure 6). The histidine kinase activity of each strain was inhibited with SYAUP-CN-26. The histidine kinase activity of the sensitive strain was 2.1338 U/g and 1.9053 U/g under 26.758 and 79.754 µg/mL treatment. Compared with the sensitive strain, the histidine kinase activity of the resistant strain increased by 23.07% and 35.61%, and the histidine kinase activities were 2.626 U/g and 2.5837 U/g, respectively.

### 2.6. Sequence Variation in the Bos1 Gene

**Figure 7:**
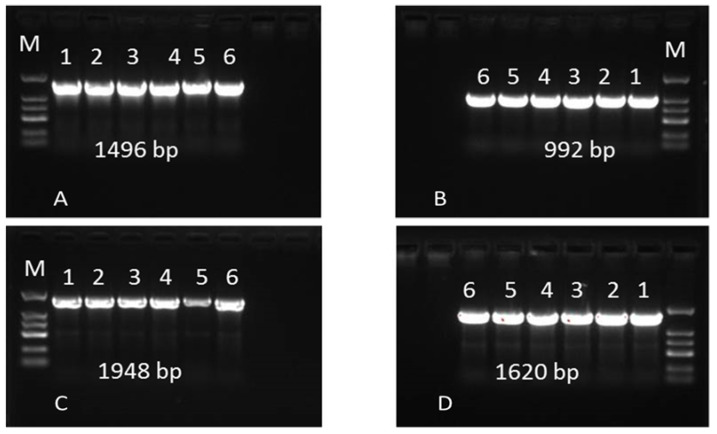
PCR amplification results of Bos1 gene fragments of *B. cinerea* sensitive and resistant strains. Note: M: DNA Maker; 1–6 were 5055, 5055-R1, 5055-R2, 5055-R3, 5055-R4, and 5055-R5, respectively. (**A**–**D**) indicate that Primer 1–4 amplified fragments.

A total of four pairs of specific primers designed by the bcos1 sequence were used to amplify the sensitive and resistant strains of *B. cinerea,* and about 1496 bp, 992 bp, 1948 bp, and 1620 bp fragments were obtained (Figure 7). After sequencing, blast comparison with the registered histidine kinase gene bos1 of *B. cinerea* in NCBI showed that sequence homology was 98%, confirming it to be the histidine kinase gene bcos1 of *B. cinerea*. Finally, the total length of the histidine kinase bcos1 gene sequence was 5675 bp, and the length of cDNA was 3948 bp, encoding 1315 amino acids. The non-conserved region was 3495 bp, encoding 696 amino acids; the conserved region was 2180 bp, encoding 619 amino acids.

By comparing the conserved base sequences, it was found that the base sequences in the conserved region of histidine kinase of sensitive strains and resistant strains were completely consistent, without any base mutation sites leading to drug resistance. One sensitive isolate and five resistant mutants were used to analyze the point mutation in the bos1 gene. The sequence alignment indicated that the four resistant strains had a single point mutation (P348L) in the amino acid sequence: proline was replaced by leucine (P348L) at position 348, but this mutation site has not been reported (Table 2). 

## 3. Discussion

Fungicides are an effective approach to controlling plant pathogenic fungi, and the mechanisms of action and resistance of fungicides play a vital role in the development and application of fungicides.

Sulfonamide compounds have always been of great interest to researchers due to their broad biological activity [30,31]. SYAUP-CN-26 is a cycloalkyl compound containing a sulfonyl group, but very few researchers have reported its mechanism of action on phytopathogens.

Maintaining ion homeostasis is a major factor in preserving the cellular energy status and membrane coupling, involving solute transport, metabolic control, and motion management. Therefore, even relatively small changes in the cell membrane might have a detrimental effect on cell metabolism and lead to cell death [32,33,34]. In vinclozolin-treated resistant strains of *B. cinerea*, after 24 h, the leakage of the sensitive strain was much higher than the resistant strain, indicating that ethylene caused a lot of lipid peroxidation in the sensitive strain cells of *Sclerotinia* [35]. In this study, SYAUP-CN-26 could damage the cell membrane of *B. cinerea*, increase its permeability, and degrade the nucleus, resulting in the leakage of fluorescent substances. However, the effect on the resistant cell membrane was not obvious, and the membrane permeability injuries caused by SYAUP-CN-26 might lead to the leakage of ions. These results were consistent with those of a previous study [25]. The ion leakage and relative conductivity of the resistant strain were lower than the sensitive strain due to little damage to the mycelial cell membrane of the resistant *B. cinerea* by SYAUP-CN-26.

In the previous studies (Table S1, Table S2 and Figure S1 in Supplementary Material), SYAUP-CN-26 had cross resistance to procymidone, belonging to dicarboximides. Therefore, it was speculated that SYAUP-CN-26 might have a similar mechanism to procymidone, leading to mycelial penetration and cell membrane lipid peroxidation [36]. Meanwhile, the results showed that SYAUP-CN-26 had a more serious effect on the synthesis of sensitive triglycerides than drug-resistant triglycerides. Triglyceride is the primary component of the cell membrane. Procimidone, possessing a similar mechanism of action with SYAUP-CN-26, could inhibit the synthesis of triglyceride in *B. cinerea* and hinder normal cell membrane formation [37], leading to the death of the sensitive strain. Furthermore, SYAUP-CN-26 significantly affected triglyceride synthesis and cell membrane structure of the sensitive and resistant strains, causing differences in resistance to SYAUP-CN-26.

These differences in the response of resistant strains to SYAUP-CN-26 might be attributed to the amino kinase gene mutation of the resistant strains. The signal regulation of the pathogen cells to adapt to the changes of osmotic pressure in the surrounding environment mainly depends on the two-component aminokinase (HK) phosphate signal transduction system. It is the core mechanism of the pathogens to feel and adapt to changes in the surrounding environment. It regulates physiological and biochemical processes, such as microbial differentiation, pathogenicity, and secondary metabolism, and increases HK activity [38]. The mutation of the amino acids in the conserved region of histidine kinase directly affects the production of drug resistance. Previous studies have cloned and sequenced the bcos1 gene [5] and found that the base sequence of the drug-resistant strain gene changes at a site of the second repeat unit, i.e., the 86th codon becomes mutated from isoleucine to serine [39]. Recent studies have also indicated that the resistance of dicarboximide fungicides is related to the mutation of the *Botrytis cinerea* bos1 gene [40,41,42]. Mutations of i365n, i365s, q369p, and n373s in the bos1 gene have been reported in many pathogens [39,43]. The present study results showed that the 348th position of the bcos1 gene was mutated from proline to leucine, which was consistent with the mutation type reported by Oshima et al. [42]. In the strains of *Botrytis cinerea* resistant to dimethamidine fungicides [44], leucine, as a regulator, participates in the regulation of intracellular signaling pathways and important biological processes, such as intracellular protein synthesis, turnover, and immune and oxidative functions [45]. It oxidizes and decomposes to produce intermediate acetyl CoA and provides ATP for other metabolic processes [46]. Therefore, the mutation of proline to leucine enhances the immune and oxidative function of bacteria, and more energy participates in metabolism, thereby enhancing resistance. Other studies have found that histidine kinase gene mutation of pathogenic bacteria is related to the generation of resistance to dimethamidine fungicides [43]. Therefore, it was inferred that the mutations in pathogen gene sequences might be associated with the production of drug resistance [44]. The study results revealed that after 10 generations of culture, the sensitivity change factor FSC value of each strain was 1.0098 and FSC ≈ 1, indicating that the resistance of these mutants to SYAUP-CN-26 could be stably inherited [33]. Therefore, it was speculated that the resistance is caused by gene mutation.

## 4. Materials and Methods

### 4.1. Fungal Isolates, Chemicals, and Culture Media

*B. cinerea* was supplied by the Plant Protection College, Shenyang Agricultural University, Shenyang, China. The concentration of the spores was adjusted to 5 × 10^5^ cfu/mL by hemocytometer.

SYAUP-CN-26 was provided by the Plant Protection College, Shenyang Agricultural University, Shenyang, China. The OMEGA Fungal DNA Extraction Kit (Solarbio Biotechnology Co., Ltd., Beijing, China), Mlbio HK Test Kit (Hailian Biotechnology Co., Ltd., Shenzhen, China), and Takara Agarose Gel DNA Purification Kit (Takara Biomedical Technology Co., Ltd., Dalian, China) were used in this study.

### 4.2. Generation of Resistant Mutants of B. cinerea to SYAUP-CN-26

The mycelial agar plugs (5 mm in diameter) were excised from 3-day-old colonies of wild-type *B. cinerea* and placed mycelia-side down on potato dextrose agar (PDA) plates containing 79.754 µg/mL (minimum inhibitory concentration, MIC) of SYAUP-CN-26 to obtain the SYAUP-CN-26-resistant mutant. After 5–7 days at 25 °C, the fast-growing mycelial tips were selected and transferred to fresh PDA plates with 100 µg/mL SYAUP-CN-26 and then placed on PDA containing 200 µg/mL SYAUP-CN-26. After 10 days at 25 °C, the fast-growing sectors were transferred to new PDA plates amended with the same SYAUP-CN-26 concentration. This step was repeated until there was no significant difference in the linear growth of the fast-growing sectors on the PDA plates with or without 200 µg/mL of SYAUP-CN-26 [47].

### 4.3. Level and Stability of Resistance of Mutants to SYAUP-CN-26

The mycelial plugs cut from the margins of 4-day-old colonies on PDA plates were placed mycelia side up on the center of the PDA plates amended with 0, 12.5, 25, 50, 100, 200 µg/mL of SYAUP-CN-26 to determine the resistance level of the mutants to SYAUP-CN-26. The EC_50_ values were calculated after 5 days. The level of resistance was represented by the resistance factor: RF = EC_50_ of mutant/EC_50_ of its parent [33].

All resistant mutants were subjected to 10 successive transfers on fungicide-free PDA plates to assess the stability of resistance to SYAUP-CN-26. RFs of the resistant mutants were determined after the first and tenth transfers, as described in the previous paragraph. The stability of resistance was represented by the FSC value (factor of sensitivity change): FSC = RF value of mutant at the1st/RF value of mutant at 10th transfers [33].

### 4.4. Effect of SYAUP-CN-26 on Cell Membrane Structure

The *B. cinerea* cultured in the PDA medium was punched to take a plate with a diameter of 5 mm. Later, three bacterial disks were transferred to 40 mL PDA medium, placed in a shaking table at 25 °C, and shaken at 120 rpm for 48 h. Afterward, SYAUP-CN-26 was added and shaken well in the PDB medium to make the concentration of SYAUP-CN-26 0, 26.758 (EC_95_), and 79.754 µg/mL (MIC). After continuous culture for 12 h, a certain amount of mycelium was randomly removed with a straw and treated with 2.5% glutaraldehyde at 4 °C for 3 h before being washed with sterile distilled water. Afterwards, the samples were transferred to a series of ethanol solutions (30%, 50%, 70%, and 90%) for 1 h, followed by 100% ethanol for 2 h. After dehydrating and embedding in Spurr’s resin, the ultrathin sections (<100 nm) were cut with a diamond knife, collected on copper 300 mesh grids, and allowed to dry on Formvar. The slices were then quickly double stained with uranyl acetate and lead citrate. Finally, the slices were prepared and visualized under an FEI Tecnai G20 Twin transmission electron microscope (FEI, Hillsboro, OR, USA) [25]. 

According to the above method, the resistant and sensitive *B. cinerea* were cultured with 0, 26.758 µg/mL (EC_95_), and 79.754 µg/mL (MIC) SYAUP-CN-26 at 120 rpm/min for 12 h. Later, the mycelium was removed, washed twice with sodium phosphate buffer (pH = 7.0), and subjected to dye with 30 µg/mL propidium iodide (PI) for 5 min. After washing the mycelium with buffer three times, the residual dye was removed, and a film was prepared and detected under a fluorescence microscope [25].

### 4.5. Measurement of Relative Electric Conductivity 

The spores of *B. cinerea* used for relative electric conductivity were collected from 15d cultures on a PDA medium. The spore suspension (5 × 10^5^ cfu/mL) was inoculated in 40 mL of PDB medium (25 °C, 120 r/min) for 36 h. The mycelium pellets were centrifuged at 5000 rpm for 10 min, washed thrice with sterilized water, and the mycelia pellets were resuspended in 50 mL of sterilized water. SYAUP-CN-26 was added to the above suspensions. The final concentrations were 26.758 µg/mL (EC_95_) and 79.754 µg/mL (MIC). The treated suspensions were incubated (25 °C, 120 r/min) for 2, 4, 8, 12, 24, and 48 h. The electric conductivity of the *B. cinerea* suspension was detected with a conductivity meter (DDS-11A; Shanghai Leici Instrument Inc., Shanghai, China). The results were expressed as the amount of relative electric conductivity (ms/cm) [25].

### 4.6. Effect of SYAUP-CN-26 on Triglyceride Synthesis (TG)

According to 2.5, the treated solutions were incubated (25 °C, 120 r/min) for 24 h. The TG contents were determined by a microplate reader using commercially available kits (Solarbio, Beijing, China), following the manufacturer’s instructions. The absorbance was measured at 420 nm [26].

### 4.7. Effect of SYAUP-CN-26 on Histidine Kinase Activity

According to 2.5, the treated solutions were incubated (25 °C, 120 r/min) for 24 h. The histidine kinase activity was determined by a microplate reader using commercially available kits (Solarbio, Beijing, China), following the manufacturer’s instructions. The absorbance was measured at 420 nm. 

### 4.8. Sequences of the Bos1 Gene in Sensitive and Resistant Strains

The genomic DNA of the sensitive and resistant strains was extracted using commercially available kits (OMEGA Fungal DNA Extraction Kit). The primers (Table 3) were designed according to the gene sequences and synthesized by Sangon Bioceth (Shanghai, China) based on the DNA sequence of bos1 (AF396827.2) of *B. cinerea*.

The amplification reactions were performed in 25 μL reaction mixture containing 12.5 µL of 2 × taq Master Mix, 2 µL of each primer (10 um), 1 μL of template DNA, and 7.5 µL of ddH_2_O. The PCR products were examined by electrophoresis in a 1.5% agarose gel in 1 × TAE buffer. The following PCR parameters were used: an initial preheating for 5 min at 95 °C; followed by 35 cycles of denaturation at 94 °C, for 30 s, annealing at 60 °C, for 30 s, and extension at 72 °C, for 4.5 min; and a final extension at 72 °C, for 10 min. PCR was conducted by a Takara Agarose Gel DNA Purification Kit (Takara Biomedical Technology Co., Ltd., Dalian, China), sequenced by the Sangon Biotech Co., Ltd. (Shanghai, China). DNAMAN software (v8.0; Lynnon Biosoft Bioinformatic Bolutions, San Ramon, CA, USA) was used to compare the amino acid sequences of the resistant mutants and sensitive strains.

### 4.9. Data Analysis

All experiments were conducted in triplicate. The statistical significance threshold (*p* < 0.05 for all analyses) was assessed by one-way ANOVA followed by Tukey’s post-hoc test for multiple comparisons using SPSS 21.0 software (SSPS Inc., Chicago, IL, USA). 

## 5. Conclusions

In this study, the mechanism of action of SYAUP-CN-26 on resistant *B. cinerea* was studied. The results showed that the effect of SYAUP-CN-26 on the resistant strain was less severe than the effects on the cell membrane structure, cell contents, relative conductivity, and production of glycerol in the sensitive strains. However, the histidine kinase (HK) activity of *B. cinerea* was more active than the sensitive strain, and proline was changed into leucine. The response of the resistant and sensitive strains to SYAUP-CN-26 was significantly different, contributing to the resistance.

## Figures and Tables

**Table 1:** Resistance level and stability of *B. cinerea* mutants to SYAUP-CN-26.

	EC50 (µg/mL)		RF		FSC
	1st	10th	1st	10th	
5055	1.6047	1.6561	--	--	
5055-R4	111.0821	115.7630	69.2230	69.9010	1.0098

**Table 2:** Comparison of Bos1 gene fragments and amino acids between *B. cinerea* sensitive and resistant strains.

AF396827.2	329	*******	348	******	368	369	370	371	372	373
	CAA	*******	CCG	******	GTC	CAG	CGC	ATG	TGG	AAC
	Q	*******	P	******	D	Q	R	M	W	N
5505	CAA	******	CCG	******	GTC	CAG	CGC	ATG	TGG	AAC
	Q	******	P	******	D	Q	R	M	W	N
5505-R1	CAA	******	CCG	******	GTC	CAG	CGC	ATG	TGG	AAC
	Q	******	P	******	D	Q	R	M	W	N
5505-R2	CAA	******	CTG	******	GTC	CAG	CGC	ATG	TGG	AAC
	Q	******	L	******	D	Q	R	M	W	N
5505-R3	CAA	******	CTG	******	GTC	CAG	CGC	ATG	TGG	AAC
	Q	******	L	******	D	Q	R	M	W	N
5505-R4	CAA	******	CTG	******	GTC	CAG	CGC	ATG	TGG	AAC
	Q	******	L	******	D	Q	R	M	W	N
5505-R5	CAA	******	CTG	******	GTC	CAG	CGC	ATG	TGG	AAC
	Q	******	L	******	D	Q	R	M	W	N

Note: ****** indicate amino acid sequence between the position 330 to 367.

**Table 3:** Primers of Bos1 in *B. cinerea* for PCR reaction.

Primers	Sequences	Annealing Temperature	Gene Sizes
F1:	AACGCGTAGCAGCTCTCGAA	62	1496
R1	ATGGTAAGATGGGTGGCCAA		
F2	GCGGGTGAAATACTCATACT	60	992
R2	ACGCTATCAAGTTCACAGAG		
F3	AACCCAAAGTCCCAATCCCAA	58	1948
R3	TTCTCGGTGGACAAGCCAA		
F4	TTACCGCGTAGATAGCTCAGTT	60	1620
R4	GCCGAAGTTCGATGATAT		

## Supplementary Materials

The following are available online [48]. Figure S1: Cross resistance of SYAUP-CN-26 to other kinds of fungicides; Table S1: Sensitivity of *B. cinerea* susceptible strains to pesticides; Table S2: Resistance of *B. cinerea* resistant mutants to pesticides.
