# Supplementary material for: Preliminary Study of Resistance Mechanism of Botrytis cinerea to SYAUP-CN-26
Source: Molecules. 2022 Jan 29;27(3):936. doi: 10.3390/molecules27030936 (PMC8839620; doi:10.3390/molecules27030936)
Supplement: Supplementary file 1 [file molecules-27-00936-s001.zip › molecules-1566840-supplementary.pdf]

Supplementary Materials

Cross resistance of resistant mutants and sensitive strains of *Botrytis cinerea* to eight insecticides. Syaup-cn-26 resistant strain and sensitive strain were cultured on PDA at 25 ℃ for 4 days, and then the plate was punched with diameter punch (Φ5 mm) and transferred to the Syaup-cn-26 PDA plate with different concentration gradients for culture:

SYAUP-CN-26: 0、12.5、25、50、100 、 200 μg/mL；

procymidone：0、0.02、0.1、0.5、2.5、12.5 μg/mL；

Isoprothiolane：0、10、20、40、80、160 μg/mL；

Phenamacril：0、20、40、80、160、320 μg/mL；

Tebuconazole：0、0.05、0.1、0.2、0.4、0.8 μg/mL；

carbendazim：0、0.01、0.05、0.25、1.25、6.25 μg/mL；

Azoxystrobin：0、0.02、0.1、0.5、2.5、12.5 μg/mL；

Pyrimethanil：0、5、10、20、40、80 μg/mL；

hymexazol：0、4、8、16、32、64 μg/mL。

Taking the EC_{50} value of syaup-cn-26 to *Botrytis cinerea* sensitive strain and resistant mutant strain as the x-axis and the EC_{50} value of another pesticides as the y-axis, the linear regression equation y=a+bX was obtained, According to the determination coefficient (R2), b value and the significance level (P value) of F test, the relationship between the toxicity of the two pesticides to *Botrytis cinerea* was analyzed: $P < 0.05$, b value was positive, indicating that there was cross resistance between the two agents, and b value was negative, indicating that there was negative cross resistance between the two pesticides. The greater R2, the stronger the correlation; $P > 0.05$, indicating that there was no cross resistance between the two agents [1].

Table S1 Sensitivity of *B. cinerea* susceptible strains to pesticides

	Regression Equation	EC_{50}（μg/mL）	*r*
SYAUP-CN-26:	Y=1.4316X+4.7331	1.6047	0.9987
procymidone	Y=1.2763X+5.6809	0.2933	0.9630
Isoprothiolane	Y=1.3485X+2.7176	49.2718	0.9919
Phenamacril	Y=2.1891X+0.5935	104.8974	0.9574
Tebuconazole	Y=1.1734X+5.9549	0.1536	0.9155
carbendazim	Y=1.8021X+7.4984	0.0611	0.9670
Azoxystrobin	Y=1.2054X+5.7844	0.2245	0.9886
Pyrimethanil	Y=0.8221X+4.4768	4.3309	0.9970
hymexazol	Y=0.9627X+3.9397	12.6343	0.9970

Table S2 Resistant of *B. cinerea* resistant mutants to pesticides

	Regression Equation	EC_{50}（μg/mL）	*r*
SYAUP-CN-26:	Y=1.2427X+2.4579	115.7630	0.9792
procymidone	Y=2.0982X+3.5168	5.1126	0.9460
Isoprothiolane	Y=1.9951X+0.9649	105.3170	0.9727
Phenamacril	Y=0.6112X+4.1332	3343.5833	0.9800
Tebuconazole	Y=1.2296X+5.5331	0.3718	0.9854
carbendazim	Y=1.6378X+6.9348	0.0927	0.9866
Azoxystrobin	Y=0.7759X+4.6242	2.2714	0.9564
Pyrimethanil	Y=0.6843X+3.7178	74.7826	0.9741
hymexazol	Y=1.2435X+3.0874	34.5292	0.9933

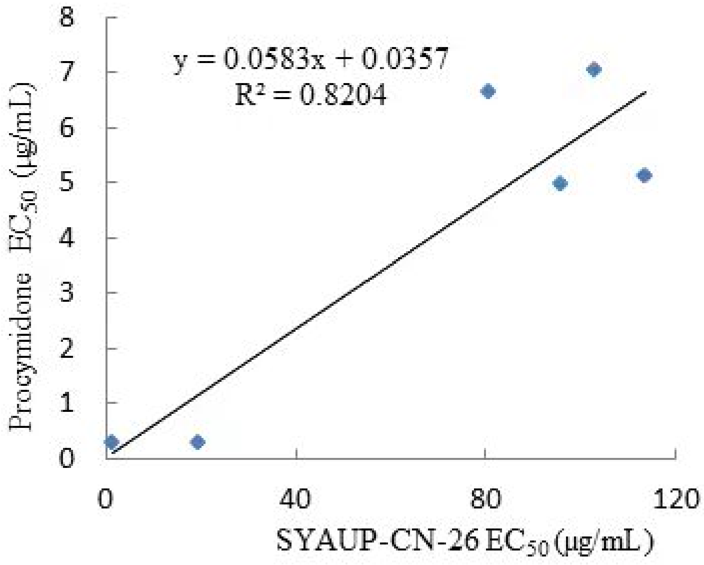

Figure S1 Cross resistance of SYAUP-CN-26 to procymidone

Taking the EC_{50} value of syaup-cn-26 to *Botrytis cinerea* strain as reference, the cross resistance of *Botrytis cinerea* resistant and sensitive to procymidone, was determined. The results showed that the regression equation of *Botrytis cinerea* to syaup-cn-26 and procymidone was $y = 0.0583x + 0.0357$, $P = 0.007 < 0.05$, the difference of F test was significant, and b value was positive, indicating that there was orthogonal mutual resistance between the sensitivity of *Botrytis cinerea* to syaup-cn-26 and procymidone, indicating that there may be a correlation between the action mechanism or resistance mechanism of syaup-cn-26 and procymidone on *Botrytis cinerea*;

Reference

1. Qi, Y.; Li, H.; Su, Y.; Zhen, W. Sensitivity to trifluzamide and main biological characteristics of resistant mutants of Rhizoctonia cerealis. *Chin. J. Pestic. Sci.* **2014**, *16*, 271–280.
